# Apgar score and hospitalization for epilepsy in childhood: a registry-based cohort study

**DOI:** 10.1186/1471-2458-6-23

**Published:** 2006-02-01

**Authors:** Vera Ehrenstein, Henrik T Sørensen, Lars Pedersen, Helle Larsen, Vibeke Holsteen, Kenneth J Rothman

**Affiliations:** 1 Department of Epidemiology, Boston University School of Public Health, Boston, MA, USA; 2 Department of Clinical Epidemiology, Aarhus University Hospital, Aarhus, Denmark; 3 Department of Gynecology and Obstetrics, Aalborg Hospital, Aalborg, Denmark; 4 Department of Pediatrics, Aalborg Hospital, Aalborg, Denmark

## Abstract

**Background:**

A depressed Apgar score at 5 minutes is a marker for perinatal insults, including neurologic damage. We examined the association between 5-minute Apgar score and the risk of epilepsy hospitalization in childhood.

**Methods:**

Using records linked from population registries, we conducted a cohort study among singleton children born alive in the period 1978–2001 in North Jutland County, Denmark. The first hospital discharge diagnosis of epilepsy during the follow-up time was the main outcome. We followed each child for up to 12 years, calculated absolute risks and risk differences, and used a Poisson regression model to estimate risk ratios for epilepsy hospitalization. We adjusted risk ratio estimates for birth weight, gestational age, mode of delivery, birth presentation, mother's age at delivery, and birth defects.

**Results:**

One percent of the 131,853 eligible newborns had a 5-minute Apgar score <7. These children were more likely to be hospitalized with epilepsy during the follow-up than were children with an Apgar score of 7 or greater. The crude risk difference for epilepsy hospitalization was 2.5 cases per 100 (95% confidence interval [CI] 1.3 to 3.8). The risk difference estimates were greater in the presence of other perinatal risk factors. The adjusted risk ratio was 2.4 (95% CI 1.5 to 3.8). Half of the 12-year risk for epilepsy hospitalization in those with a depressed Apgar score occurred during the first year of life. The risk ratio during the first year of life was 4.9 (95% CI 2.0 to 12.3).

**Conclusion:**

An Apgar score <7 at five minutes predicts an increase in the subsequent risk of epilepsy hospitalization. This association is amplified by other perinatal risk factors.

## Background

Designed to assess infants' condition immediately after birth, Apgar score [1] is a cumulative ranking of five clinical signs – heart rate, respiratory effort, muscle tone, reflex activity, and color – each assigned a rating of 0, 1, or 2 with lower number corresponding to poorer condition [2]. Apgar scores take on integer values from zero to ten and are measured at one and five minutes of age. A prolonged Apgar score below four is a component of a diagnosis of asphyxia and is a stronger predictor of neonatal death than the pH of umbilical artery blood [3,4]. A depressed five-minute Apgar score reflects a host of intrauterine and perinatal insults, some of which are also known or suspected risk factors for neurologic morbidity: hypoxic and mechanical brain trauma, birth defects, non-optimal birth weight or gestation, breech presentation, delivery complications, maternal age and smoking, as well as the newborn's poor response to resuscitation prompted by a low one-minute Apgar score [3-13]. Unknown prenatal causes of neurologic damage (e. g., subclinical in-utero infection [14]) may likewise contribute to the value of the Apgar score, making it a sign of increased general vulnerability of the infant.

The five-minute Apgar score correlates better with subsequent neurologic morbidity than the one-minute score [3]. Studies report associations of five-minute Apgar score with cerebral palsy, mental retardation, seizures, and with minor neurologic disability [15,16]. The association of Apgar score with epilepsy – one of the most prevalent neurologic disorders [17] – was reported by a single study, in which epilepsy was not the primary outcome [11]. Moreover, the statistical analysis was inappropriate for the varying follow-up, and modification of the effect of the Apgar score by other perinatal characteristics was not addressed.

Using data from Danish population registries, we conducted a cohort study to examine the relation between five-minute Apgar score and the risk of hospitalization for epilepsy. We also examined whether this relation depended on perinatal characteristics that are known or suspected risk factors for neurologic morbidity.

## Methods

### Study population and design

We conducted the study in the Birth Cohort of North Jutland County, Denmark, using routinely collected electronically stored data from the Danish Medical Birth Registry, North Jutland County Hospital Discharge Registry, and the Danish Civil Registration System [18]. In the Birth Registry, we identified all single live births from 1978 through 2001 and retrieved variables for five-minute Apgar score, birth weight, gestational age, mode of delivery, birth presentation, birth defects (defined here as malformations discovered during the birth hospitalization), mother's age at delivery, and mother's smoking in pregnancy.

From the Hospital Discharge Registry, we retrieved records of epilepsy hospitalizations. We used the International Classification of Diseases version 8 (ICD-8) codes 345.00–345.99 (before 1994), and ICD-10 codes G40.0-G40.9, G41.0-G41.9 (thereafter) to identify epilepsy cases. Whenever available, we also retrieved records on epilepsy hospitalizations for mothers and fathers of the newborns.

Data on emigration and death were from the Civil Registration System. Records were linked using the National Civil Registration number, which is a unique identifier assigned to all Danish residents at birth and used in all public records. The follow-up time for each child was calculated from birth until the date of the first epilepsy hospitalization, emigration, death, 12th birthday, or December 31, 2002.

The informed consent was not required for this study, since it was conducted using public-domain records with the identifier removed from the analysis dataset.

### Data analysis

From the incidence of epilepsy, we estimated the corresponding risk from birth to age 12 and calculated the risk difference associated with a depressed five-minute Apgar score, defined as a score below seven. We examined the extent to which the risk and risk difference varied according to birth weight in grams (≤ 2500, 2501–3000, 3001–3500, 3501–4000, ≥ 4001), gestational age in weeks (<28, 28 – 36, 37 – 42, >42), mode of delivery (spontaneous, assisted by vacuum or forceps, caesarean), birth presentation (cephalic vs. non-cephalic), birth defects (present/absent), mother's age at delivery in years (≤ 20, 21–30, ≥ 31 years), and when available, dichotomous variables for mother's smoking during pregnancy and parental epilepsy hospitalization.

We used Poisson regression [19,20] to model the rate of epilepsy hospitalization and to estimate the risk ratio, while adjusting simultaneously for the effects of non-cephalic birth presentation, birth weight, gestational age, maternal age, birth defects, and mode of delivery. Maternal smoking in pregnancy became reportable to the Birth Registry after 1990. We repeated the adjusted analysis in a subcohort of children born after 1990, with a variable for maternal smoking in pregnancy added into the model. The Hospital Discharge Registry was established in 1977 and thus contained only partial information on parental hospitalizations for our cohort. We estimated that the earliest parental hospitalizations would be recorded in the Hospital Discharge Registry for children who were born after 1994 and did the regression analysis separately for this subcohort, with an indicator variable for parental epilepsy hospitalization added to the model.

For 69 randomly selected children hospitalized with epilepsy in 1998–2000, we compared Hospital Discharge Registry records with paper medical records in order to estimate positive predictive value of the registered discharge diagnosis. For the paper records, we defined an epilepsy case as a physician-recorded epilepsy diagnosis, based on two or more unprovoked seizure episodes or on electroencephalography findings, or both [21]. Febrile seizures were excluded.

We analyzed the data with version 8.02 of SAS^® ^software (SAS Institute, Cary, NC).

## Results

From the 132,932 neonates who had records in the Birth Registry and met our entry criteria, we excluded 1,079 (0.8%) with a missing five-minute Apgar score. Of the remaining 131,853 newborns, 476 (0.4%) had a five-minute Apgar score below four, 847 (0.6%) had Apgar scores between four and six; the rest of the newborns had Apgar scores of seven or above. Table 1 shows prevalence of depressed Apgar score according to perinatal characteristics. Infants with low birth weight, short gestation, non-cephalic birth presentation, non-spontaneous delivery, birth defects, and notably, a parent who had been hospitalized for epilepsy, were more likely to have five-minute Apgar score below seven compared with the cohort as a whole.

There were 815 cases of epilepsy hospitalization, corresponding to a 12-year risk of 0.8% (Table 2). Twenty-seven cases occurred among those with five-minute Apgar score below seven, including eight cases among those with Apgar score below four. The latter group had the shortest median follow-up time of 8.4 years.

Table 3 shows risks and risk differences related to having a depressed Apgar score in categories of perinatal characteristics. Risks were consistently greater in children with Apgar scores below seven compared with children with Apgar score of seven or greater. The overall excess risk related to having a depressed Apgar score was 2.5 cases per 100 persons (95% confidence interval [CI] 1.3 to 3.8 cases per 100 persons). The absolute risk increase was greater among children with either low or elevated birth weight (respectively, 4.5 and 3.2 per 100); birth defects (4.2 per 100); maternal smoking in pregnancy (5.3 per 100), gestation beyond 42 weeks (18.6 per 100); and a history of parental epilepsy hospitalization (36.1 per 100), though the latter two estimates were based on few cases.

Risks of epilepsy hospitalization in Apgar score categories 0–3 and 4–6 were similar (Table 2) and the categories were combined for the further analyses. The crude risk ratio for epilepsy hospitalization was 4.3 (95% CI 2.0 to 6.3) for an Apgar score below seven vs. an Apgar score of seven and above, and the adjusted risk ratio was 2.4 (95% CI 1.5 to 3.8).

In the subcohort of infants born in 1991–2001 with added maternal smoking information, the adjusted risk ratio was 3.8 (95% CI 1.9 to 7.5), and in the subcohort of births with added information on parental hospitalization for epilepsy (1995–2001), the adjusted risk ratio was 5.2 (95% CI 2.1 to 13.0) (Table 4). Removing maternal smoking or parental epilepsy variables, or both, from these analyses of the restricted cohorts, however, did not substantially change the adjusted estimates, suggesting that larger risk ratio estimates resulted from the subcohort having a shorter follow-up rather than from better control of confounding. Adjusted risk ratios for epilepsy hospitalization were 1.6 (95% CI 1.1 to 2.5) for maternal smoking in pregnancy and 1.8 (95% CI 0.6 to 5.8) for having a parent hospitalized with the disease.

Half of the epilepsy hospitalizations among those with Apgar score below seven occurred during the first year of life. Restricting the analysis to that period yielded an adjusted risk ratio estimate of 4.9 (95% CI 2.0 to 12.3).

The epilepsy diagnosis validation of the 69 cases recorded in the Hospital Discharge Registry showed that 52 of them also had a diagnosis of epilepsy recorded in the paper chart. Of the 17 unconfirmed epilepsy diagnoses, two were coding errors; five were seizures without a definite diagnosis of epilepsy; five were suspected seizures; one was asphyxia; one was mental retardation; one was an unspecified neurologic problem; and two were heart failure diagnoses. Thus, while 75 percent of validated cases fulfilled strict clinical criteria for epilepsy, a further seven to 14 percent had seizures without being given an epilepsy diagnosis. Coding errors occurred in three percent of the examined records. None of the children with epilepsy whose diagnose was validated had a depressed five-minute Apgar score.

Compared with the analysis cohort, the small (<1%) group of infants with a missing 5-minute Apgar score had a lower median birth weight, higher prevalence of birth defects, and were more likely to be in a non-cephalic birth presentation. The risk of epilepsy among them was 0.6 percent (6 cases). Under the hypothetical extreme assumption that all these newborns actually had a 5-minute Apgar score below seven, the 12-year risk of epilepsy hospitalization in the exposed group would have decreased slightly but would still be about twice the risk among infants with Apgar score of seven or greater. Such an extreme distribution of missing Apgar score values would of course be unlikely, given their observed distribution in the analysis cohort and median follow-up time of 12 years.

## Discussion

In this large population-based study with prospectively collected data, having a depressed five-minute Apgar score was consistently associated with increased risk of epilepsy hospitalization in the first 12 years of life. It is often noted that the overwhelming majority of babies with a depressed Apgar score grow up healthy [3,15]. Nevertheless, the two- to four-fold increase in the risk of epilepsy hospitalization that we found is substantial. We observed a greater absolute effect of Apgar score on risk of epilepsy hospitalization among children delivered with the assistance of forceps or a vacuum extractor. The absolute effect was also amplified by having a low birth weight, and by maternal smoking in pregnancy. These characteristics alone were not strong risk factors for epilepsy in our data, but combined with a depressed Apgar score, predicted a large increase in risk. This finding is consistent with the current opinion that epilepsy can result from the gradual accumulation of environmental insults to the central nervous system [17].

The risk of epilepsy hospitalization was somewhat greater among babies with Apgar scores between four and six than in babies with scores below four. We offer two possible explanations for this observation. First, because babies with a low Apgar score face a high mortality, epilepsy and death are for them competing outcomes and some children will not survive long enough to develop epilepsy [22]. We obtained mortality data for babies born in North Jutland County in 1980–2001 and found that 30% of the newborns with a five-minute Apgar score below four died within the first year of life, compared with 14% and 0.4% among those with scores of 4–6 and 7–10. Second, epilepsy due to perinatal complications is likely to have an early onset. We found that all epilepsy cases occurring among those who fell into the lowest Apgar score group (0–3) were diagnosed before the age of six. Between ages 6 and 12, these children had zero risk of epilepsy in these data, contributing to a comparatively low 12-year risk estimate in this group.

The association between perinatal history and neurologic morbidity has been shown in a number of studies: low birth weight and prematurity are risk factors for neonatal seizures [5]; in-utero nicotine exposure has been implicated in occurrence of cerebral hemorrhage [6]; breech presentation affects cognitive function [10]; and inadequate intrauterine growth increases risk of cerebral palsy [7]. We found that the association between depressed Apgar score and epilepsy remained strong even after removing the effect of low birth weight, preterm and postterm birth, birth defects, non-spontaneous delivery, and non-cephalic birth presentation.

The outcome of interest of this study was a diagnosis of epilepsy that resulted in hospitalization. Not all children diagnosed with epilepsy are hospitalized, and the risk of epilepsy diagnosed among outpatients may exhibit a different relation to five-minute Apgar score. Registration of outpatient visits in North Jutland County started after 1993. Based on a portion of these data, we estimate that about 20 percent of epilepsy diagnoses are made among outpatients, with an incidence of 3/1000 person-years for those with Apgar score below 7 and 0.2/1000 person-years among those with Apgar score of 7 and above. Based on 32 outpatient epilepsy cases observed in these data, we estimated the adjusted risk ratio for outpatient epilepsy to be 9.8 (95% CI 2.6 to 36.6) over six years of follow-up.

Epilepsy develops by a number of mechanisms, many still unknown [17,23,24] and its association with Apgar score may or may not reflect a causal connection. Insofar as the value of five-minute Apgar score is a rough composite measure of neurologic vulnerability, it may reflect the action of a set of prenatal and perinatal factors that cause epilepsy or increase individual's susceptibility to developing it. The stronger associations seen for shorter follow-up times support the notion of the importance of perinatal factors in determining epilepsy risk in early childhood.

Danish Birth Registry data have been validated and found to have high quality [25]. Hospital discharge diagnoses, however, are not always accurate [26]. Our validation of a small sample of cases suggests that roughly 25% of epilepsy records in the hospital discharge registry do not correspond to strict epilepsy diagnoses; this proportion of false-positive diagnoses is an important limitation of these data. Validated ascertainment of all cases was not logistically possible for the countywide long-term data used here. Since birth data are entered before and independently of discharge data, however, the rate of false positive diagnoses are not likely to differ much by Apgar score, unless the conditions that constitute the false positive cases are themselves related to Apgar score [27].

Registry data inherently lack clinical detail. Thus, we did not have information on head trauma or neonatal seizures – important precursors of epilepsy [17,28,29]. Nevertheless, the complex causal constellations for both Apgar score and epilepsy suggest that these are unlikely to entirely explain away the observed association. The ability to differentiate between elective and emergency caesarean delivery and between different types of non-cephalic birth presentations would elucidate the role of these characteristics in affecting neurologic morbidity and in determining the predictive value of the Apgar score.

## Conclusion

We found that neither prematurity nor low birth weight was associated with epilepsy hospitalization as strongly as was a low Apgar score. The Apgar score, which is an easily and routinely collected correlate of a host of perinatal events, may be a useful addition to birth weight and gestational age in predicting epilepsy morbidity among infants.

## Competing interests

The author(s) declare that they have no competing interests.

## Authors' contributions

VE participated in designing the study, analyzed the data, and lead the writing; HTS conceived the study, oversaw its conduct, data acquisition, and contributed to interpretation of results and manuscript writing; LP prepared the dataset and participated in data analysis; HL carried out outcome validation and contributed to drafting the manuscript and interpreting results; VH provided clinical expertise and helped draft the manuscript and interpret results; KJR participated in study design, contributed to manuscript writing, data analysis and interpretation. All authors read and approved the final manuscript.

## Pre-publication history

The pre-publication history for this paper can be accessed here:



## Figures and Tables

**Table 1:** Birth characteristics and 5-minute Apgar score of 131,853 Danish newborns.

Characteristic	N	Five-minute Apgar score < 7	
		
		Frequency	Prevalence, %
Entire cohort	131,853	1,323	1.0
Birth weight			
≤2500 g	5,633	334	5.9
2501–3000 g	17,043	163	1.0
3001–3500 g	44,381	289	0.6
3501–4000 g	43,779	296	0.7
≥4001 g	20,860	165	0.8
No record	157	76	48.4
Gestational age			
<28 weeks	179	55	30.7
28–36 weeks	5,258	265	5.0
37–42 weeks	116,527	847	0.7
>42 weeks	3,866	45	1.2
No record	6,023	111	1.8
Mode of delivery			
Spontaneous	106,449	708	0.7
Assisted	10,007	213	2.1
Caesarean	15,350	401	2.6
No record	47	1	2.1
Birth presentation			
Cephalic	109,724	813	0.7
All other	14,837	420	2.8
No record	7,292	90	1.2
Any birth defect			
Absent	122,274	1,087	0.9
Present	9,579	236	2.5
Mother's age at delivery			
≤20 years	7,509	87	1.2
21–30 years	89,986	879	1.0
≥31 years	34,358	357	1.0
Mother smoked in pregnancy*			
No	43,375	485	1.1
Yes	17,804	191	1.1
No record	1,620	45	2.8
Parental epilepsy hospitalization^#^			
No	38,332	356	0.9
Yes	439	8	1.8

*Births after 1990, N = 62,799. ^#^Births after 1994, N = 38,771.

**Table 2:** Incidence of epilepsy hospitalization by 5-minute Apgar score.

	Five-minute Apgar score			Total
		
	0–3	4–6	7–10	
Cases	8	19	788	815
Total births	476	847	130,530	131,853
Median years of follow-up	8.4	9.8	12.0	12.0
Person-years	3,193	6,559	1,215,045	1,224,797
Incidence, per 1000 person-years	2.5	2.9	0.6	0.7
12-year risk, per 100 persons	3.5	3.5	0.8	0.8

**Table 3:** Risks and risk differences for epilepsy hospitalization according to 5-minute Apgar score and other characteristics.

Characteristic	Risk per 100 persons (no. of cases)			Risk difference, cases per100 (95% CI)
		
	Overall	Apgar score <7	Apgar score ≥ 7, reference	
Entire cohort	0.8 (815)	3.3 (27)	0.8 (788)	2.5 (1.3 to 3.8)
Birth weight				
≤2500 g	1.7 (70)	3.2 (5)	1.6 (65)	1.6 (-1.2 to 4.5)
2501–3000 g	1.0 (133)	5.5 (6)	0.9 (127)	4.5 (0.1 to 8.9)
3001–3500 g	0.8 (275)	3.7 (7)	0.8 (268)	2.9 (0.2 to 5.6)
3501–4000 g	0.7 (228)	1.4 (3)	0.7 (225)	0.8 (-0.9 to 2.4)
≥4001 g	0.7 (106)	3.8 (4)	0.7 (102)	3.2 (-0.6 to 6.9)
Gestational age				
<28 weeks	1.6 (1)	0 (0)	1.9 (1)	-1.9 (-5.5 to 1.8)
28–36 weeks	1.4 (55)	2.9 (4)	1.3 (51)	1.6 (-1.3 to 4.4)
37–42 weeks	0.7 (684)	2.5 (14)	0.7 (670)	1.7 (0.4 to 3.0)
>42 weeks	0.9 (20)	19.3 (4)	0.7 (16)	18.6 (-0.3 to 37.6)
Mode of delivery				
Spontaneous	0.7 (621)	3.0 (13)	0.7 (608)	2.2 (0.6 to 3.9)
Assisted	0.9 (68)	4.4 (6)	0.8 (62)	3.6 (0.1 to 7.1)
Caesarean	1.1 (125)	2.9 (7)	1.1 (118)	1.9 (-0.3 to 4.0)
Birth presentation				
Cephalic	0.7 (662)	3.0 (16)	0.7 (646)	2.3 (0.8 to 3.8)
All other	1.1 (129)	3.7 (9)	1.1 (120)	2.6 (0.2 to 5.0)
Birth defects				
Absent	0.7 (639)	2.8 (20)	0.7 (619)	2.2 (0.9 to 3.4)
Present	2.3 (176)	6.5 (7)	2.2 (169)	4.2 (-0.6 to 9.0)
Mother's age at delivery				
≤20 years	1.1 (72)	3.4 (2)	1.1 (70)	2.3 (-2.4 to 7.1)
21–30 years	0.8 (572)	3.1 (17)	0.8 (555)	2.3 (0.9 to 3.8)
≥31 years	0.7 (171)	3.9 (8)	0.7 (163)	3.2 (0.5 to 5.9)
Mother smoked in pregnancy*				
No	0.6 (141)	2.4 (6)	0.6 (135)	1.8 (-0.1 to 3.7)
Yes	1.0 (99)	6.3 (6)	0.9 (93)	5.3 (0.3 to 10.4)
Parental epilepsy hospitalization^#^				
No	0.8 (122)	5.9 (7)	0.8 (115)	5.1 (0.7 to 9.4)
Yes	1.9 (3)	37.4 (1)	1.3 (2)	36.1 (-37.1 to 109.4)

* Births after 1990, N = 62,799. ^# ^Births after 1994, N = 38,771.

**Table 4:** Crude and adjusted risk ratios for epilepsy hospitalization.

Analysis cohort	N	Risk ratio for 5-minute Apgar score<7 (95% CI)	
		
		Crude	Adjusted
All births (up to 12 years of follow-up)	131,853 (815 cases)	4.3 (2.0 to 6.3)	2.4 (1.5 to 3.8)*
All births with follow-up restricted to the first year of life	131,853 (217 cases)	8.4 (4.9 to 14.4)	4.9 (2.0 to 12.3)*
Births 1991–2001 (maternal smoking data complete)	62,799 (249 cases)	4.9 (2.8 to 8.7)	3.8 (1.9 to 7.5)^#^
Births 1995–2002 (maternal smoking and parental epilepsy data complete)	38,771 (125 cases)	8.1 (4.0 to 16.7)	5.2 (2.1 to 13.0)^†^

* Adjusted for birth weight, gestational age, mode of delivery, birth presentation, mother's age at delivery, and birth defects.

^# ^Adjusted for all of the above plus maternal smoking.

^† ^Adjusted for all of the above plus parental epilepsy.
